# Decreasing Lens Irradiation on Brain Imaging: A Multi-CT Scanner Quality Improvement Project

**DOI:** 10.7759/cureus.47357

**Published:** 2023-10-20

**Authors:** Berk Abay, James C Sankeshwar, Hussein Kamel

**Affiliations:** 1 General Surgery, Barts Health NHS Trust, London, GBR; 2 Radiology, Barts Health NHS Trust, London, GBR

**Keywords:** lens exclusion, ionising radiation exposure, lens irradiation, brain imaging, cataracts

## Abstract

Aims: Cataracts, a leading global cause of blindness, are associated with ionising radiation exposure. This audit aimed to enhance lens exclusion during non-contrast head computed tomography (CT) scans at Newham University Hospital (NUH) using two CT scanners.

Methods: A retrospective audit of non-contrast head CT scans at NUH excluded scans for trauma and imaging of orbital structures. A one-week audit in April 2023 assessed lens exclusion, compared to the Royal College of Radiologists (RCR) standards. A total of 101 consecutive scans were analysed and 63 (62%) scans were included in the final study. Thirty-eight percent of the scans were excluded according to the exclusion criteria of head, neck and facial traumas, orbital infections and papilledema. Results were presented, followed by a three-month radiographer re-education period, emphasizing gantry tilt and patient positioning. A reaudit in August 2023 evaluated outcomes. For the reaudit, 183 consecutive scans were analysed, with 131 (72%) scans included in the final study and 52 (28%) scans excluded according to the same exclusion criteria as the first audit.

Results: Lens exclusion in non-contrast head CT scans improved significantly from 0/63 (0%) compliance to 19/131 (14.50%) (p=0005) compliance with the standards. Variability in radiographer practices, 'near misses' and time constraints were identified as challenges. Staff turnover impacted compliance.

Conclusion: This audit has shed light on a critical aspect of patient care in the field of radiology. This research underscores the importance of rigorous and standardised protocols in radiological procedures, particularly when it comes to protecting the lens of the eye. By enhancing lens exclusion during non-contrast head CT scans, we have taken a significant step in mitigating the risk associated with ionising radiation exposure. Although substantial improvements were made, achieving the RCR audit standard remained elusive. Ongoing re-education, reaudits and a multidisciplinary approach are necessary to optimise radiographer adherence and reduce ionising radiation exposure to the lens during head CT scans. This quality improvement project proves that continued emphasis on gantry tilt and patient positioning in radiographer education and training can make a significant difference in patient safety. As we move forward, let us remember that even small improvements can make a big difference in safeguarding the health and well-being of patients.

## Introduction

Cataracts represent a significant global cause of blindness, with a staggering 94 million individuals estimated to have experienced visual impairment due to cataracts, as reported by the World Health Organization (WHO) in their August 2023 publication [1]. Numerous extensive population-based studies have consistently revealed a positive correlation between age and the prevalence of cataracts, ranging from 3.9% in those aged 55-64 years to a striking 92.6% among individuals aged 80 years and older [2-4]. Furthermore, research has identified ionising radiation exposure as a noteworthy risk factor in the development of cataracts [5,6]. Effective methods for minimising lens irradiation involve implementing a 10-12 degree gantry tilt above the orbito-meatal line in computed tomography (CT) scans or encouraging patients to adopt a chin-tuck position [7]. Within Newham University Hospital (NUH), two CT scanners, namely, the SOMATOM Definition Flash (Siemens Healthineers, Germany) without gantry tilt and Aquilion ONE / PRISM Edition (Canon Medical Systems, Japan) with gantry tilt capabilities of +/-30 degrees, are currently in use. This quality improvement project aims to reduce the proportion of non-contrast head CT scans that inadvertently expose the lens to ionising radiation.

## Materials and methods

**Figure 1:**
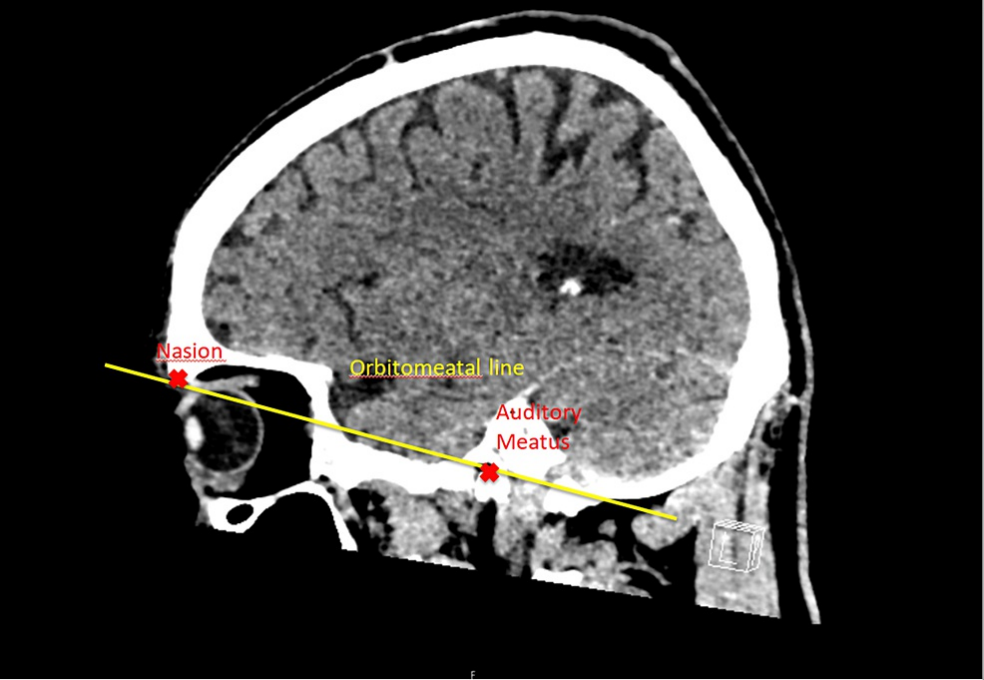
Orbitomeatal line

This study represents a comprehensive retrospective analysis of non-contrast head CT scans conducted on patients of all age groups at NUH. A total of 101 scans were reviewed for the first audit consisting of 46 women (46%) and 55 men (54%) in April 2023. The reaudit included a total of 183 scans with 88 women (48%) and 95 men (52%) in August 2023. The primary objective of this quality improvement project was to evaluate the extent to which lens exclusion, a critical safety measure in radiological practice, was effectively implemented during these head CT scans. Two CT scanners were included in this study: SOMATOM Definition Flash without gantry tilt and Aquilion ONE / PRISM Edition with +/-30 degrees of gantry tilt. Gantry tilt allows angled scanning and helps avoid direct exposure to the radiosensitive organs like the lens [8]. Figure 1 shows the orbitomeatal line where the gantry should be positioned to lower the irradiation of the lens.

Analysis of 101 consecutive head CT scans from a one-week period in April 2023 were audited for lens exclusion by the authors, under supervision of a consultant radiologist. Eighty (79%) and 21 (21%) scans were analysed with the SOMATOM and Aquilion scanners, respectively. Scans on patients with head, neck and facial traumas, orbital infections and papilledema were excluded from the study as these scans were performed for imaging the orbits, paranasal sinuses, base of the skull or cervical spine, and it would have been impossible to exclude the lens from the irradiated area. A total of 38 scans (38%) were excluded from the first audit, and 52 scans (28%) were excluded from the reaudit according to the previously mentioned exclusion criteria. Table 1 shows the number of patients excluded according to each criteria from both audits. The results obtained from this evaluation were subsequently compared against the professional guidelines set forth by the Royal College of Radiologists (RCR) audit standard, which stipulates a target of achieving 100% lens exclusion [9].

**Table 1:** Number of patients excluded according to each exclusion criteria from the initial audit and reaudit

	Initial audit (April 2023) (n=101)		Reaudit (August 2023) (n=183)	
n (%)	SOMATOM Definition Flash n=80 (79)	Aquilion ONE / PRISM Edition n=21 (21)	SOMATOM Definition Flash n=147 (80)	Aquilion ONE / PRISM Edition n=36 (20)
Head trauma n (%)	18 (22.5)	4 (19)	20 (13.6)	6 (16.7)
Neck trauma n (%)	3 (3.8)	0 (0)	6 (4.1)	0 (0)
Facial trauma n (%)	10 (12.5)	2 (9.5)	15 (10)	3 (8.3)
Orbital infection n (%)	1 (1.3)	0 (0)	1 (0.7)	0 (0)
Papilledema n (%)	0 (0)	0 (0)	1 (0.7)	0 (0)
Excluded n (%)	32 (40)	6 (28.6)	43 (29)	9 (25)
Included n (%)	48 (60)	15 (71.4)	104 (71)	27 (75)

It is worth mentioning that this audit was conducted with the formal approval of the hospital's clinical effectiveness unit, ensuring ethical and methodological integrity. The clinical effectiveness unit at NUH has approved the audit and the reaudit with the identification numbers of 13,374 and 13,533, respectively. To disseminate the findings and raise awareness among relevant stakeholders, the results of this audit were presented at both the hospital radiology governance meeting and the trust radiographers meeting. Furthermore, an educational initiative was initiated over a span of three months, focusing on the radiographers responsible for performing these scans. The educational efforts centered on emphasizing the critical importance of lens exclusion from the irradiated area. Specific recommendations were provided, including the incorporation of gantry tilt for the Aquilion ONE / PRISM Edition scans and the positioning of patients in a chin-tuck posture for the SOMATOM Definition Flash scans. The audit was replicated over a one-week period in August 2023 to assess the impact of the educational intervention and measure any improvements achieved. A total of 147 (80%) and 36 (20%) scans were collected with the SOMATOM and Aquilion CT scanners, respectively. Statistical analysis was conducted using Fisher's exact tests to determine the independence of categorical variables and independent t-tests to compare means across groups. The tests were considered statistically significant if p-value was <0.05.

## Results

Table 2 provides a detailed overview of the patient demographics and the rates of lens exclusion observed in both the initial audit and the subsequent reaudit. For the initial audit, the mean age of the patients was 57.35 years with a standard deviation of 22.27, while for the reaudit, the mean age was slightly lower at 53.08 years with a standard deviation of 23.36. It is worth noting that both audits encompassed a wide age range, spanning from six to 97 years, demonstrating the diverse patient population under examination. Sex distribution and mean age were found to be similar between the two audit cohorts, indicating a consistent patient demographic profile.

**Table 2:** Patient demographics and lens exclusion compared in both audits.

	Initial audit (April 2023)	Reaudit (August 2023)	p-values
Total scans reviewed	n = 101	n = 183	
Excluded scans according to the exclusion criteria	n = 38	n = 52	
Total scans included in the audit	n = 63	n = 131	
Sex (Female %)	46/101 (46%)	88/183 (48%)	p=0.7
Age (years ± SD)	57.35 ± 22.27	53.08 ± 23.36	p=0.1355
Age range	6, 97	1, 93	
Lens exclusion overall (%)	0/63 (0%)	19/131 (14.5%)	p=0.0005
Exclusion of lenses on each CT scanner (%)			
SOMATOM Definition Flash	0/48 (0%)	13/104 (12.5%)	p=0.01
Aquilion ONE / PRISM Edition	0/15 (0%)	6/27 (22.2%)	p=0.07

**Figure 2:**
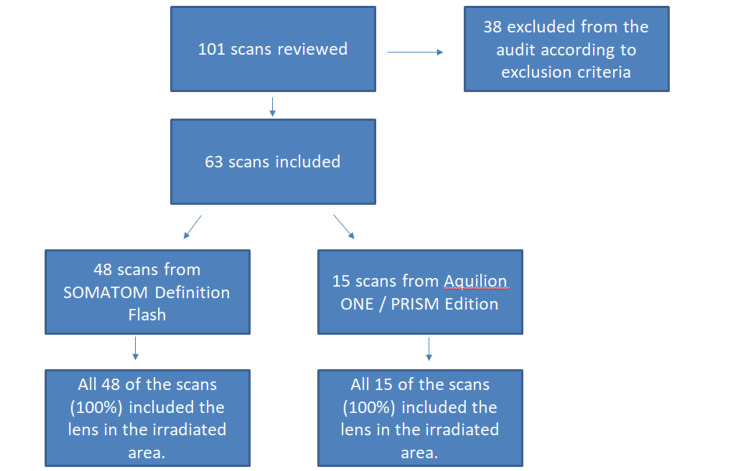
Included scans and results of the first audit

In the first audit, a total of 101 scans were assessed, with 63 scans meeting the inclusion criteria. Notably, the initial audit identified a concerning issue: all 48 scans conducted using the SOMATOM Definition Flash CT scanner and all 15 scans employing the Aquilion ONE / PRISM Edition CT scanner included the lens within the irradiated area. This complete lack of lens exclusion indicated a striking 0% compliance with the RCR standards, highlighting a significant safety concern. Figure 2 shows the included and excluded scans with each of the scanners.

Following the three-month period of radiographer reeducation, the reaudit was conducted in August 2023, involving a larger dataset of 183 reviewed scans, with 131 meeting the inclusion criteria. In this reaudit, 13 out of 104 scans involving the SOMATOM Definition Flash CT scanner successfully excluded the lens. This outcome was statistically significant (p=0.01), indicating that the educational program implemented for radiographers had a demonstrable impact on improving lens exclusion for this specific scanner. However, for the Aquilion CT scanner, although there was an improvement with six out of 27 scans excluding the lens, statistical significance was not achieved (p=0.07).

To summarize the outcomes, the improvement in lens exclusion for non-contrast head CT scans conducted with the SOMATOM Definition Flash CT scanner increased significantly from 0% to 12.5% (p=0.01), while for the Aquilion ONE / PRISM Edition CT scanner, there was an improvement from 0% to 22.2% (p=0.07). When considering the overall improvement across both scanners, it amounted to a notable 14.50% enhancement, which was statistically significant (p=0.0005). These findings underscore the effectiveness of the reeducation program in fostering safer radiological practices and ensuring compliance with critical safety standards.

## Discussion

In the present study, we aimed to address the critical issue of ionising radiation exposure to the lens during non-contrast head CT scans, which has been linked to an increased risk of cataract formation. Our audit at NUH focused on improving lens exclusion during these scans and compared our results with the RCR audit standards, which advocate for 100% lens exclusion [9].

It is well known in the literature that ionising radiation increases the risk for cataracts formation in the future for both the patient and the radiographer [10,11]. According to the RCR, all brain scans should be performed with the baseline set to exclude the lens from the irradiated area. Moreover, European recommendations recommend the utilisation of a CT gantry tilt ranging from 10 to 12 degrees above the orbitomeatal line whenever it is accessible [12].

To our information, there are only two other articles that were recently published in 2023 that suggest that lens exclusion can help with reducing lens irradiation and cataracts formation [7,13]. Our article differs from these articles by incorporating one scanner with a gantry tilt and one without, enabling us to see the difference between the scanners. The gantry tilt has demonstrated its effectiveness in significantly diminishing the radiation dose delivered to the lens, as evidenced by Tarkiainen et al.'s findings. Successful exclusion of lens was increased to 11.8% from 1.8% when the gantry tilt was used in 98% of the cases [13]. Our study shows that in the absence of a gantry tilt, the chin-tuck position can also be a comparable alternative as evidenced by the lens exclusion increasing to 12.5% from 0% in our study.

Lens dose reduction varies from 9% to 30% in the literature [14-16]. In a comprehensive retrospective analysis, the estimated lens dose was recorded at 25.9 mGy (with a range of 17.8-49.2 mGy) when the eyes were included within the scan field, in contrast to a substantially lower dose of 1.5 mGy (with a range of 0.4-1.9 mGy) when the eyes were deliberately excluded through the use of a gantry tilt [13]. Although Poon et al. [6] reported that a gantry tilt is the most effective way to reduce lens dose in CT scans, the chin-tuck position can be used in machines lacking the ability of a gantry tilt with significant numbers of lenses excluded as evidenced by our study.

The risk is exponentially greater for patients who receive repeat CT scans. Yuan et al. reported that as the number of CT scans increased, the adjusted hazard ratio of cataract incidence also increased. Patients with no CT scans were used as referent and their adjusted hazard ratio was 1. Patients with one to two CT scans that included the lens had an adjusted hazard ratio of 1.61, whereas patients with five or more CT scans that included the lens had an adjusted hazard ratio of 2.12 [5]. It is possible that as patients age, they go through more CT scans, and if the lens is included in the irradiated area, the cataract formation risk increases with each scan. The implementation of a gantry tilt along the supraorbital margin to exclude the lens is also recommended as a standard practice as it was found to be highly effective for reducing radiation dose outcomes on CT scans [6]. 

At the end of our reaudit, there was a significant improvement in lens exclusion with the gantry tilt and chin-tuck position, but it did not reach the RCR audit standard. The RCR audit standard was elusive as it recommends 100% compliance with excluding the lens, which could prove difficult in the real-world clinical practice. We identified three possible reasons for this.

First, the practice between radiographers varied, which can be seen in other institutions as well [17]. Some radiographers exhibited enthusiasm in implementing the recommended changes, while others displayed more caution. Some also raised concerns about the higher radiation dose associated with a gantry tilt, approximately 50% higher. While this is a valid concern, it is imperative to acknowledge that excluding the lens from the irradiated area significantly reduces radiation exposure to this radiosensitive organ. The decision to prioritize the protection of radiosensitive organs against the increment in radiation levels should be assessed on a benefit-risk scale and could prove to be beneficial.

Second, during the scan reviews, we encountered several 'near misses' where the lenses were included in the irradiated area by a margin of 1 cm or less. This suggests that adapting to the practice of correctly positioning the gantry or the patient may require some time, especially considering that this is a relatively new practice in our hospital.

Lastly, a limited number of scans were conducted using the Aquilion ONE / PRISM Edition scanner, resulting in a small sample size for this particular machine, thereby reducing the statistical power of the audit for that particular machine. The majority of scans were performed on the SOMATOM Definition Flash, which lacks gantry tilt capability. In addition, this scanner serves patients presenting to the emergency department and a substantial backlog of patients awaiting scans, placing time constraints on radiographers. In such circumstances, it can be challenging for radiographers to consistently position every patient to exclude the lens. Furthermore, NUH has experienced increased staff turnover in various departments, including radiology, which can impede the achievement of audit standards [18]. Notably, a prior audit conducted in our hospital revealed no improvement in lens exclusion. To enhance the overall compliance rates of 12.5% and 22.2% achieved in this audit, the department plans to conduct regular re-education sessions and implement subsequent audit cycles to further improve radiographer adherence to safety protocols.

## Conclusions

This audit has shed light on a critical aspect of patient care in the field of radiology. This research underscores the importance of rigorous and standardised protocols in radiological procedures, particularly when it comes to protecting the lens of the eye. By enhancing lens exclusion during non-contrast head CT scans, we have taken a significant step in mitigating the risk associated with ionising radiation exposure. Although substantial improvements were made, achieving the RCR audit standard remained elusive. Ongoing re-education, reaudits and a multidisciplinary approach are necessary to optimise radiographer adherence and reduce ionising radiation exposure to the lens during head CT scans. This quality improvement project proves that continued emphasis on the gantry tilt and patient positioning in radiographer education and training can make a significant difference in patient safety. As we move forward, let us remember that even small improvements can make a big difference in safeguarding the health and well-being of patients.
